# Quantitative assessment of DNA damage directly in lens epithelial cells from senile cataract patients

**Published:** 2011-01-05

**Authors:** K. Sorte, P. Sune, A. Bhake, V.B. Shivkumar, N. Gangane, A. Basak

**Affiliations:** 1 Department of Biochemistry, Jawaharlal Nehru Medical College, Maharashtra, India; 2 Department of Ophthalmology, Jawaharlal Nehru Medical College, Maharashtra, India; 3 Department of Pathology, Jawaharlal Nehru Medical College, Maharashtra, India; 4 Department of Pathology, Mahatma Gandhi Institute of Medical Sciences, Maharashtra, India

## Abstract

**Purpose:**

Most of the studies regarding DNA damage in lens epithelial cells (LECs) of cataract patients have been done on lymphocyte or human LECs cultures. Studies of DNA damage directly in LECs of cataract patients are scanty and, to our knowledge, neither photographic evidence nor has a quantitative assessment of DNA damage have been put forward. In our study, we assessed and quantified DNA damage directly in the LECs of senile cataract patients, right after cataract surgery.

**Methods:**

LECs were taken from different morphologic types of senile cataract patients after surgery and DNA damage was immediately assessed by comet assay. Quantitative assessment of DNA damage was conducted using CometScore ™ software.

**Results:**

There were no prominent comets in most of the LECs of the control subjects, but comets were found in cataractous LECs. DNA fragments in the tail of the comet gave smearing (not banded), which was indicative of chemical damage (i.e., not site specific). DNA damage in the LECs of cataract patients was highly significant (p<0.001). DNA damage in cortical cataracts was significant (p<0.01) when compared to that of nuclear or posterior subcapsular cataracts, but the DNA damage between nuclear and posterior subcapsular cataracts was not significant. Furthermore, we found disrupted nuclear membranes in some of the nuclei in LECs of patients, but not in the control subjects.

**Conclusions:**

In senile cataract patients, LECs DNA was randomly damaged and this type of damage was possible by reactive oxygen species (ROS). The damage was found maximally in the cortical type of cataracts. Oxidative DNA damage of the LECs may be one of the etiology of senile cataractogenesis.

## Introduction

The lens epithelium cell (LEC) is the center of metabolic activities in lenses [1]. Oxidative damage to LECs plays a significant role in the pathogenesis of many forms of cataracts [2,3]. The eyes are continuously exposed to UV radiation [4] and exposure to UV-rays causes oxidative stress to generate reactive oxygen species (ROS), which leads to DNA damage [5-7].

Extensive damage to DNA and membrane pumps, by ROS, occurs, followed by loss of epithelial cell viability and death by necrotic and apoptotic mechanism in cataractogenesis [2]. This damage occurs when the levels of pro-oxidants (ROS and other free radicals) exceed the ability of the cell to respond through antioxidant defense and, ultimately, leads to modifications of proteins, damage to lipids and DNA, and to cell death [8,9]. These cause lens opacity. Nucleic acids are prone to oxidative damage by ROS. Continuous attack of ROS results in unscheduled DNA synthesis in LECs that undergo apoptosis [5].The marker of DNA oxidation, 8-hydroxyguanine (OHG), was found to be increased in a normal human LEC culture after induced oxidative stress [10]. DNA excision, destruction, and fragmentation have been observed, following free radicals attack on DNA [8]. Irradiation of mice heads produced oxidative stress and DNA damage in LECs [11]. One important study showed association of DNA damage with human cataracts but, unfortunately, photographic evidence of bovine DNA damage was presented [12]. Moreover, the extent of DNA damage was arbitrarily assessed. Most of the studies regarding DNA damage had focused on lymphocytes or on human LEC cultures rather than directly on LECs from senile cataract patients [3,13,14].

Therefore, these studies did not simulate in vivo metabolism of the LECs of cataract patients. Extrapolation of the results of animal studies or human LEC cultures to humans is to be done cautiously. In our study, we quantitated DNA damage directly in LECs from three different types of senile cataract patients just after cataract surgery.

## Methods

### Procedure to remove the lens anterior capsule

#### From healthy control subjects

After removing the cornea, the anterior capsule was removed using a forceps giving continuous curvilinear capsulorhexis (CCC). Thirteen samples were collected during the study period. Patients ranged in age from 60 to 80 years. The sample size is small due to the unavailability of healthy subjects.

#### From senile cataract patients

**Figure 1:**
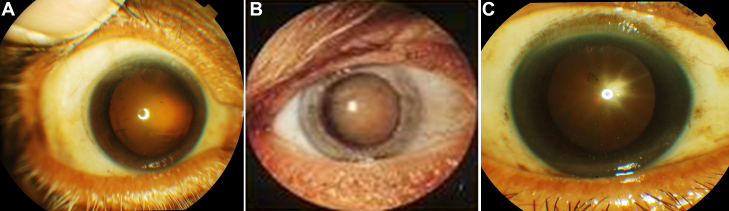
Appearance of lenses in patients. **A**: Cortical cataract. **B**: Nuclear cataract. **C**: Posterior subcapsular cataract.

Under local anesthesia (2% xylocaine), through a clear corneal incision (3 mm in length), continuous curvilinear capsulorhexis (CCC) was done. The anterior capsule was removed with viscoexpression through clear corneal incision and the anterior capsule was collected using forceps. This was done to avoid direct damage to the LECs. A total of 49 samples were collected from the 50–90 year patient age group. Of these, 28 samples were cortical cataracts, 12 were nuclear cataracts and nine were posterior subcapsular types of senile cataracts. After removal of the anterior capsule, the sample was immediately kept in minimum essential media and transported to the laboratory from the Ophthalmology operation theater. Photographs of three different types of senile cataract patients’ eyes were presented (Figure 1A-C).

### Sample preservation

A single rhexis was placed in Eagle’s Minimal Essential Medium (MEM), containing 10% fetal calf serum, and incubated in a 5% CO_2_ incubator at 37 °C until the samples were processed [15]. Maximum time lapse from sample collection to starting of processing was 30 min.

### LEC viability testing

**Figure 2:**
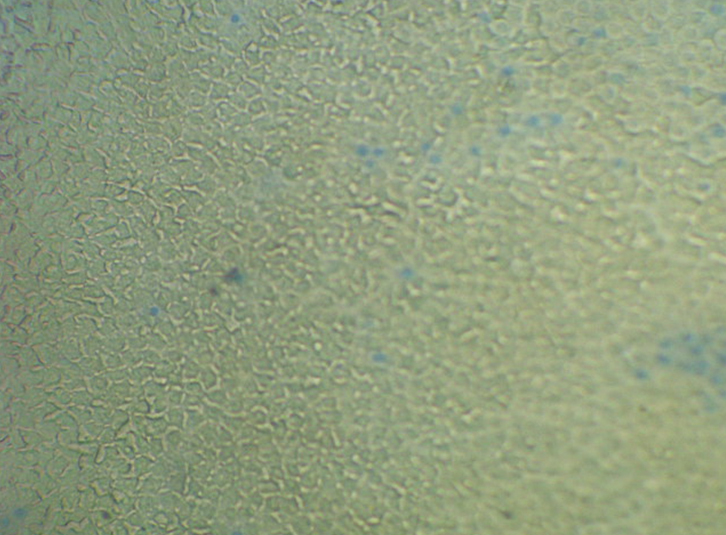
Viability test (trypan blue exclusion) of LECs (10×) in a senile cataract patient. Blue staining indicates dead cells.

Before preparation for the comet assay, LEC viability was checked using the trypan blue exclusion test [16]. All the samples collected were viable (Figure 2). The blue color indicates dead cells.

### Sample preparation

Cell suspension of the LECs was prepared using mechanical shaking of the capsule (in 50 µl of PBS), by hand, for 10–15 min at 4 °C to shed LECs from the lens capsule. After this, the capsule was discarded. Cell suspension of the LECs was used for the comet assay (single cell gel electrophoresis) to assess the nature and extent of the DNA damage.

Comet assay was conducted, according to the procedure developed by Singh et al. [17], with the following modifications: 1) Embedding of cells in the second gel layer by mixing equal volumes of cell suspension of 50 µl with 2% LMPA, instead of 80 µl of 1% LMPA and 20 µl of cell suspension; and 2) lyses of LECs were done at 4 °C for 8 h instead of 2 h.

After staining, the slides were examined under fluorescent microscope. Digital photographs were captured and DNA damage was assessed using CometScore™ software.

## Results

**Figure 3:**
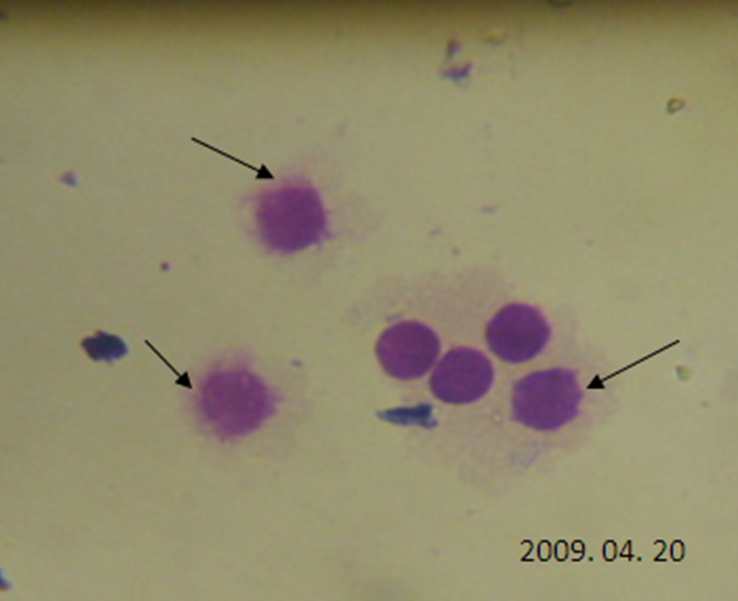
Nuclei of Lens epithelial cells (100×) from a senile cataract patient. The arrow shows damaged nuclear margins.

**Figure 4:**
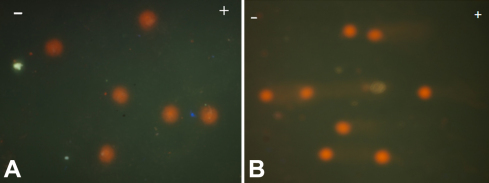
Comet assay in LECs. **A**: Control subject. **B**: Senile cataract patient.

**Figure 5:**
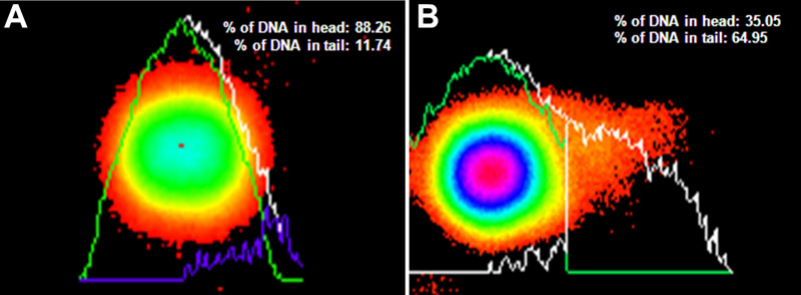
DNA damage quantification by software in the head and tail of a comet in LEC. **A**: Control subject. **B**: Senile cataract patient.

**Figure 6:**
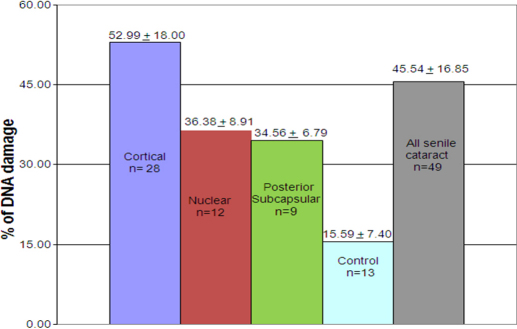
Percent of DNA damage in LECs of different morphological types in senile cataract patients and control subjects.

Few LEC nuclear membranes were found to be disrupted in cataract patients (shown by arrow, stained with H&E; Figure 3). No prominent comets were found in most of the LECs of the control subjects (Figure 4A), but comets were found in most of the cataractous LECs (Figure 4B). Quantization of DNA damage in comets was shown for both the control patients (Figure 5A) and the cataract patients (Figure 5B). The percentage of DNA damage in the tail of the comets was: 15.59±7.40 for the control subjects (Table 1) and 45.54±16.85 for the overall cataract patients. The difference was significant (p<0.001). The percentage of DNA damage in cortical, nuclear and posterior subcapsular cataracts was: 52.99±18.00, 36.38±8.91, and 34.56±6.79, respectively (Table 2, Table 3, and Table 4, and Figure 6). Maximum and minimum % DNA in the comet tail was found to be 88.80 and 18.34 in the cataract patients and 24.81 and 1.60 in the control subjects. DNA damage in cortical cataracts was significantly higher when compared to the DNA damage found in patients with nuclear cataracts (p<0.01) and patients with posterior subcapsular cataracts (p<0.01), but no significant difference was found between nuclear and posterior subcapsular cataract patients (p>0.10; Table 5).

**Table 1:** Percent of DNA damage in LECs in control subjects.

**Control sample number**	**Head (% DNA)**	**Tail (% DNA)**
1	88.26	11.74
2	75.56	24.44
3	85.04	14.36
4	86.04	13.34
5	75.75	24.25
6	75.19	24.81
7	81.78	18.22
8	80.2	19.80
9	84.03	15.97
10	84.12	15.88
11	97.67	2.33
12	98.4	1.60
13	84.02	15.98
Mean	84.31	15.59
SD.	±7.37	±7.40

Maximum DNA in tail was found to be 24.81% and minimum was 1.60%.

**Table 2:** Percent DNA damage in LECs in Cortical Senile Cataract patients.

**Sample number**	**Head (% DNA)**	**Tail (% DNA)**
1	62.4	37.60
2	18.79	81.21
3	75.91	24.09
4	37.56	62.44
5	36.55	63.45
6	68.45	31.55
7	35.05	64.95
8	32.9	67.10
9	45.01	54.99
10	32.33	67.67
11	39.44	60.56
12	54.9	45.10
13	68.2	31.80
14	11.2	88.80
15	54.2	45.80
16	15.04	84.06
17	37.96	62.04
18	40.01	59.99
19	54.99	45.01
20	70.63	29.37
21	36.55	63.45
22	32.9	67.10
23	77.43	22.57
24	65.44	34.56
25	48.99	51.01
26	39.89	60.11
27	59.78	40.22
28	62.78	37.22
Mean	46.97	52.99
SD.	±18.06	±18.00

Maximum DNA in the tail was found to be 84.06% and the minimum was 22.57%.

**Table 3:** Percent of DNA damage in LECs in Nuclear Senile Cataract patients.

**Sample number**	**Head (% DNA)**	**Tail (% DNA)**
1	71.66	28.34
2	68.88	31.12
3	68.9	31.10
4	61.34	38.66
5	71.28	28.72
6	81.66	28.34
7	68.52	31.48
8	68.32	31.68
9	56.9	43.10
10	60.41	39.51
11	44.78	55.22
12	50.67	49.33
Mean	64.44	36.38
SD.	±10.12	±8.91

The maximum DNA in the tail was found to be 55.22% and the minimum was 28.34%.

**Table 4:** Percent of DNA damage in LECs in Posterior Subcapsular Senile Cataract patients.

**Sample number**	**Head (% DNA)**	**Tail (% DNA)**
1	70.63	29.37
2	69.22	30.78
3	64.45	35.55
4	68.53	31.47
5	64.97	35.03
6	56.57	43.43
7	52.88	47.12
8	67.53	32.47
9	74.14	25.86
Mean	65.44	34.56
SD.	±6.79	±6.79

The maximum DNA in the tail was found to be 47.12% and the minimum was 25.86%.

**Table 5:** Statistical analysis of the percentage of DNA damage in different morphological types in Senile Cataract patients and Control subjects.

**Study Group**	**‘t’ value**	**p value**	**Significance**
Cataract patients versus control subjects	6.222	<0.001	Highly significant
Cortical cataract versus Nuclear cataract	3.025	<0.01	Significant
Cortical cataract versus Posterior subcapsular cataract	2.980	<0.01	Significant
Nuclear cataract versus Posterior subcapsular cataract	0.511	>0.10	Not significant

## Discussion

The extent of DNA damage can be assessed by 8-hydroxyguanine assay and comet assay. In comet assay, the tail showed smearing of DNA fragments instead of bands, which was indicative of random damage (i.e., non-enzymatic). This is possible with ROS, which acts by chemical reaction. Oxidative damage to the DNA of LECs, and increased unscheduled expression of genes, are both responsible for apoptosis, which is one of the important etiologies of cataractogenesis [18]. Guanine residues of DNA are chemically modified by OH**˙** to form 8-hydroxyguanine, thus DNA damage takes place by direct attack of the ROS. Aggregation of high molecular weight (HMW) proteins, like histone due to disulfide bond formation [19], is evidenced because –SH groups are highly susceptible to H_2_O_2_ [20]. Several evidences indicate that the post-translational modifications of proteins and oxidation of membrane lipids, due to ROS, are preceded by HMW protein aggregation that buries the functional group and, ultimately, leads to cataract formation [21,22]. DNA damage was observed after induced microwave radiation [14] and soft X-ray radiation in mice LECs [11]. DNA damage was also found in human LEC cultures with induced oxidative stress by hydrogen peroxide [3,23].

We found a variable percentage of DNA damage in LECs of senile cataract patients and in few healthy subjects. DNA damage in cataract patients was found to be highly significant (Table 2, Table 3, and Figure 5). To our surprise, we found few disrupted nuclear membranes in the LECs of cataract patients (Figure 3). Our findings of DNA damage in the LECs of cataract patients confirmed the earlier observation, but extent of DNA damage assay was arbitrary and, unfortunately, no photographic evidence of DNA damage of human LECs was presented [12]. We have presented photographs of DNA damage in the LECs of both control subjects and senile cataract patients.

Therefore, in senile cataract patients, LECs DNA was randomly damaged and this type of damage was possible by ROS. The maximal damage was found in the cortical type of cataracts. Oxidative DNA damage of the LECs may be one of the etiologies of senile cataractogenesis.
